# Differential release of extracellular vesicle tRNA from oxidative stressed renal cells and ischemic kidneys

**DOI:** 10.1038/s41598-022-05648-3

**Published:** 2022-01-31

**Authors:** Hee Kyung Lee, Byung Rho Lee, Tae Jin Lee, Chang Min Lee, Chenglong Li, Paul M. O’Connor, Zheng Dong, Sang-Ho Kwon

**Affiliations:** 1 Department of Cellular Biology and Anatomy, Medical College of Georgia, Augusta University, Augusta, GA USA; 2 Center for Biotechnology and Genomic Medicine, Medical College of Georgia, Augusta University, Augusta, GA USA; 3 Department of Physiology, Medical College of Georgia, Augusta University, Augusta, GA USA; 4 Charlie Norwood VA Medical Center, Augusta, GA USA

**Keywords:** Cell biology, Molecular medicine, Nephrology

## Abstract

While urine-based liquid biopsy has expanded to the analyses of extracellular nucleic acids, the potential of transfer RNA (tRNA) encapsulated within extracellular vesicles has not been explored as a new class of urine biomarkers for kidney injury. Using rat kidney and mouse tubular cell injury models, we tested if extracellular vesicle-loaded tRNA and their m^1^A (*N*^1^-methyladenosine) modification reflect oxidative stress of kidney injury and determined the mechanism of tRNA packaging into extracellular vesicles. We determined a set of extracellular vesicle-loaded, isoaccepting tRNAs differentially released after ischemia–reperfusion injury and oxidative stress. Next, we found that m^1^A modification of extracellular vesicle tRNAs, despite an increase of the methylated tRNAs in intracellular vesicles, showed little or no change under oxidative stress. Mechanistically, oxidative stress decreases tRNA loading into intracellular vesicles while the tRNA-loaded vesicles are accumulated due to decreased release of the vesicles from the cell surface. Furthermore, Maf1-mediated transcriptional repression of the tRNAs decreases the cargo availability for extracellular vesicle release in response to oxidative stress. Taken together, our data support that release of extracellular vesicle tRNAs reflects oxidative stress of kidney tubules which might be useful to detect ischemic kidney injury and could lead to rebalance protein translation under oxidative stress.

## Introduction

Acute kidney injury (AKI) is characterized by the rapid decline in kidney function and associated with morbidity, mortality, and health care costs^1, 2^. Unfortunately, a positive secular trend in the incidence rate of AKI has been observed in both hospitalized and non-hospitalized patient populations^3^. Furthermore, incomplete recovery from AKI leads to long-term kidney diseases^4^. To date, treatments for AKI are highly limited except supportive care. Given the current situation, the best opportunity for improving the outcome likely lies in accurate and timely diagnosis. Diagnosis criteria for AKI have relied on changes in serum creatinine (srCr) and urine output because these readouts can reflect kidney function, assuming that impaired renal function indicates renal injury. Yet, srCr is also dependent on extrarenal factors and slowly responds to kidney injury, which has made intensive searches for other markers directly representing cellular injury/damage^5–8^.

Extracellular vesicles in urine originate from the epithelia aligned in the nephron segments and urogenital tract^9^. Because of the sizes of urinary vesicles^10^, it is unlikely extracellular vesicles from blood freely pass the fine pore sizes of the slit diaphragm and the barrier of the glomerular basement membrane. Hence, utilizing urinary vesicles as a source of biomarkers has an advantage to gain the information from kidneys, which might be useful to detect kidney injury. Furthermore, extracellular vesicles have shown to mediate intercellular communication in kidney injury^11, 12^. Urinary vesicles carry various RNAs, including tRNA, rRNA, scaRNA, snoRNA, snRNA, piRNA, mRNA, and miRNA^13^. Of note, mRNA and miRNA appear to be a main research focus as their expression/abundance is regulated in response to cellular states, not like other RNA species with housekeeping functions. Indeed, highly diverse miRNA and mRNA sequences were loaded in extracellular vesicles^13, 14^. However, mRNA and miRNA are relatively scant in urinary vesicles when compared with rRNA and tRNA^13–15^.

tRNAs are ubiquitous small non-coding RNAs that link the genetic information transcribed in mRNA to protein translation^16^. They have long been considered as a simple adaptor molecule in protein synthesis delivering amino acids to translating ribosomes. Many recent studies, however, began to reveal the noncanonical function of tRNAs, particularly in stress signaling^17^. Under stress, tRNAs undergo dynamic expression^18^, base modification^19, 20^, fragmentation^21, 22^, and nuclear compartmentalization^23^, all of which can affect the rate and fidelity of protein translation. These findings suggest tRNAs participate in active role for stress signaling. Given the abundance of tRNAs in urinary vesicles as well as stress-regulated tRNA biogenesis, we here asked whether cellular stress alters transport of tRNAs to the extracellular space through vesicle traffic and thus extracellular release of tRNA packaged in vesicles might report injured kidney state.

## Results

### Identification of tRNAs differentially released in urinary vesicles from kidney injury

**Figure 1:**
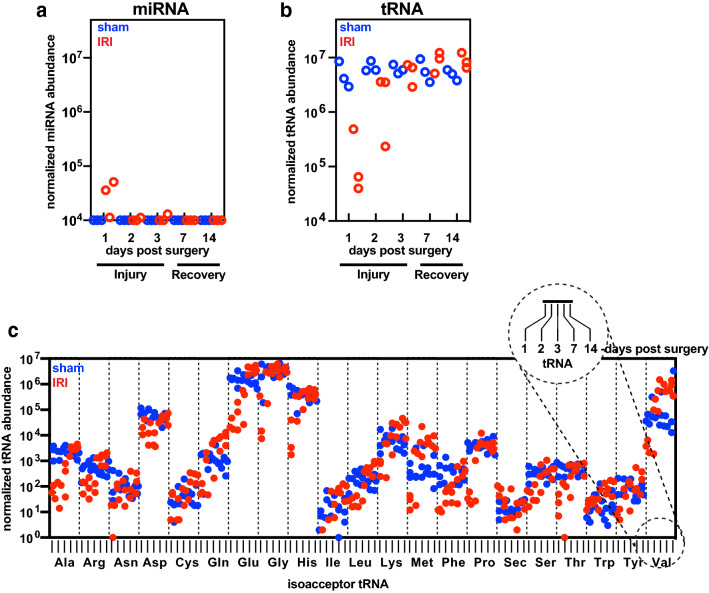
Identification of isoaccepting tRNAs differentially released in extracellular vesicles of urine from kidney injury. (**a** and **b**) Interleaved scatter plots showing expression levels of the indicated RNA species, miRNA (**a**) and tRNA (**b**) in urinary extracellular vesicles from sham (blue) or injured (red) animals during the time course of renal ischemia/reperfusion injury (IRI). The states of injury and recovery were determined based on the levels of serum creatinine and urea nitrogen. The dataset used for this re-analysis was previously published in GEO database (GPL18694) and our publication^14^. All read counts were normalized using DEseq2 package. (**c**) A scatter plot showing normalized expression levels of isoacceptor tRNAs in urinary extracellular vesicles from sham (blue) and IRI (red) at the indicated days following renal ischemia/reperfusion injury. Alphabet letters denote the 21 genetically-encoded amino acids. All read counts were normalized using DEseq2 package.

To examine the global relationship between the abundance of tRNAs in urinary vesicles and the progression of acute kidney injury, we re-analyzed our previous small RNA-sequencing data^14^ and determined changes in abundance of tRNAs released through urinary vesicles during an extended period of time following renal ischemic injury (Fig. 1). The presence of a significant fraction of urinary vesicle tRNAs in mapped sequence read counts^14^ led us to test the hypothesis that the levels of tRNAs in urinary extracellular vesicle might reflect acute kidney injury. Based on the levels of serum creatinine and blood urea nitrogen measured in the study, we previously determined day 1-to-day 3 as injury state and day 7-to-day 14 as recovery state^14^, and the indicated states were marked in the plots accordingly. Total miRNAs released from injured animals via urinary vesicles were increased at 1 day after renal injury and were thereafter back to the basal levels, when compared with those from sham-operated animals (Fig. 1a). In sharp contrast, tRNAs in extracellular vesicles of urine from injured animals remarkably decreased at the injury state and in turn, gradually increased to the levels similar to those of sham animals during the recovery state (Fig. 1b). Taken together, this data suggests that urinary excretion of tRNAs via extracellular vesicles, including exosomes is differentially controlled in respond to renal injury states.

We next focused on the longitudinal expression of individual tRNA (Fig. 1c). Each isoacceptor tRNA, which is charged with the same amino acid and has a different nucleotide sequences, showed vastly different abundance in urinary vesicles, ranging from the normalized count of 1 to 10^7^. For example, tRNA^Glu^ and tRNA^Gly^ were the two most abundant tRNAs in urinary vesicles whereas tRNA^Ile^ and tRNA^sec^ were the least abundant ones. Most isoaccepting tRNAs showed a prominent reduction in extracellular vesicles at the early injury whereas isoaccepting tRNA^Trp^ and tRNA^Sec^ showed a subtle increase, compared with the sham operated group. Whether this increased abundance of these relatively infrequent tRNAs is significant still remains to be determined, due to their relative low sequencing counts. Convergence behavior of expression of tRNAs in sham and injured group over time was further shown through Jensen-Shannon divergence measurements of individual tRNA abundance over the time course of 14 days (Supplemental Fig. S1).

### Validation of tRNAs differentially released in extracellular vesicles from renal ischemia/reperfusion injury

Utilizing extracellular vesicles, including exosomes, could greatly help rigorous and reproducible quantification in RNA abundance because it decreases the complexity of RNA abundance in biological fluid samples. Given the observed high abundance of tRNAs in urine extracellular vesicles and their dramatic abundance changes in response to the renal injury, we chose extracellular vesicle-loaded tRNA^Ala^, tRNA^Glu^, tRNA^Asp^, and tRNA^Val^ for further validation. Unlike most other cytoplasmic unstable RNAs, tRNA has a stabilized tertiary structure with numerous post-transcriptional modifications, including methylation^24^. This structure is known to interfere with cDNA synthesis, which could result in a biased quantitation from RNA-sequencing (RNA-seq) and real-time polymerase chain reaction (qPCR). To relax the stable structure of tRNAs and hence to lower amplification bias, partial hydrolysis with alkaline condition or demethylation was recently applied to the cytoplasmic tRNA profiling field^25–27^. Because our results were from a conventional small RNA-seq, and furthermore little is known about the posttranscriptional modification status of urinary vesicle-loaded tRNAs, we compared several approaches to optimize cDNA synthesis with urinary vesicle tRNAs, and thereby hoping to achieve accurate quantification (Supplemental Fig. S2). Posttranscriptional methylation of tRNAs in urinary vesicles, if present, was eliminated by *E.coli* demethylases prior to reverse transcription reaction. Two commonly used priming methods with random-hexamer and oligo d(T) were also compared to test the primer access to tRNAs for qPCR analysis. For oligo d(T) priming, poly (A) tail was added with *E.coli* poly (A) polymerase to deacylated tRNAs, and thus provides oligo d(T) priming to the extended tRNAs for reverse transcription with d(T)_23_VN, an anchored oligo d(T). Reverse transcription of extracellular vesicle RNAs without poly (A) adenylation, primed with d(T)_23_VN, an anchored oligo d(T) was used as negative control. As the same starting material was used in this experiment, a lower Cq, quantitation cycle value indicates a more efficient reverse transcription. The Cq from demethylated and poly (A)-tailed tRNAs showed a remarkable downward shift, compared with the other methods (Supplemental Fig. S2a and c). This result shows that reverse transcription with demethylated, poly (A)-extended vesicular tRNAs give rise to a significant amplification for a better quantification. It is also consistent that tRNAs in urine vesicles are methylated though the degree of modification between these cytoplasmic and vesicular RNAs remains undetermined in this experiment. Next, these three conditions were used to determine the abundance of extracellular vesicle tRNAs from urine samples from three different animals per group. RT-qPCR analysis showed similar relative expression of tRNAs but the variability was significantly reduced when urinary vesicle tRNA were demethylated and primed with d(T)_23_VN (Supplemental Fig. S2b) and thus we decided to use demethylation and polyadenylation steps for qPCR-based tRNA quantification.

**Figure 2:**
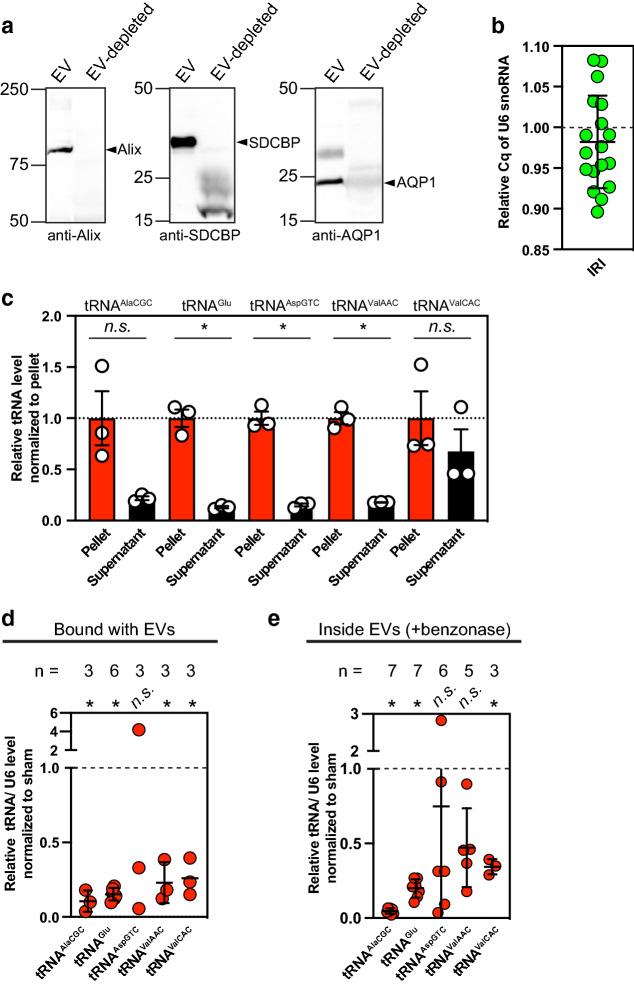
Identification of tRNAs in extracellular vesicles from urine and validation of tRNAs differentially released in extracellular vesicles from renal ischemia/reperfusion injury (IRI). (**a**) Immunoblotting analysis showing the indicated proteins in extracellular vesicle (EV) fraction. As a control experiment, resuspended extracellular vesicles were spun at 200 K × g to produce extracellular vesicle (EV)-depleted condition. (**b**) U6 snoRNA of urinary extracellular vesicles as an adequate reference gene in this experimental condition. Note that the Cq distributions of U6 from urinary extracellular vesicles in IRI were not statistically different. Shown are relative distributions of quantification cycle, Cq of U6 snoRNA from extracellular vesicles of IRI urine normalized to that of sham urine (volume). All urine samples were collected from 24 h after IRI surgery. (**c**) Among urinary tRNAs with decreased expression 24 h after IRI compared with those from sham, tRNA^Ala^, tRNA^Glu^, tRNA^Asp^, and tRNA^ValAAC^, but not tRNA^ValCAC^ are mainly present in 200 K × g pellets. The bar graph shows the relative levels of the indicated tRNAs from rat urine either in the pellets or supernatants from 200 K × g ultracentrifugation. *n.s.* statistically non-significant; *p < 0.05. (**d**) Relative abundance of urinary tRNAs from renal IRI that are associated with urinary extracellular vesicles fractionation. Expression levels of the indicated tRNA were normalized using U6 snoRNA within each group, and then relative expression values of tRNA of IRI group normalized to those of sham group were plotted. EV, extracellular vesicles. *n.s.* statistically non-significant; *p < 0.05. (**e**) Relative abundance of benzonase-resistant, urinary extracellular vesicle tRNAs from renal IRI. Expression levels of the indicated tRNA were normalized using U6 snoRNA within each group, and then relative expression values of tRNA of IRI group normalized to those of sham group were plotted. Note that benzonase was used to measure the abundance of indicated tRNAs which are mostly present inside EVs. EVs, extracellular vesicles. *n.s.* statistically non-significant; *p < 0.05.

Immunoblotting analysis showed that extracellular vesicle markers^14^, including Syntenin (also called SDCBP), Alix, and Aquaporin 1 were enriched in the extracellular vesicle fraction, but not in the extracellular vesicle-depleted fraction from urine, validating the extracellular vesicle isolation protocol used in this study (Fig. 2a). With this optimized experimental setting, we tested if U6 snoRNA (U6) can serve as a reference gene to normalize the expression of urinary vesicle tRNAs (Fig. 2b). Bilateral renal ischemia/ reperfusion surgery was performed as previously described^28^ and then extracellular vesicles were isolated from urine samples collected from either sham or animals 24-h after renal ischemia–reperfusion injury (IRI). A single time point of a 24-h period was chosen as it was the earliest increase of serum creatinine values measured in the experimental setting. The RNAs in extracellular vesicles were then extracted to quantify U6 snoRNA. The quantitative distribution of relative Cq of U6 snoRNA from IRI, which is normalized to sham indicates that the expression of U6 snoRNA in urine vesicles remains unchanged 24-h post IRI, and thereby we decided to use U6 snoRNA to control for error in normalizing tRNA expression. With U6 snoRNA as a reference, we performed RT-qPCR on the pellet and supernatant fractions from 200,000 × g spin to determine where the injury-responsive extracellular tRNAs are detected. After spun, the extracellular tRNAs were predominantly enriched in the pellets that contain extracellular vesicles (Fig. 2c and Supplemental Fig. S2c).

Next, to test if these tRNAs are bound to the outside of extracellular vesicles or encapsulated in vesicles, we treated isolated vesicles with benzonase nuclease which is used to degrade all forms of DNAs and RNAs. RT-qPCR analysis of the candidate set of urinary tRNA^Ala^, tRNA^Glu^, tRNA^Asp^, and tRNA^Val^ from sham and injured rats confirmed the changes in tRNA abundance observed in the RNA-seq analysis (Fig. 2d). Additionally, the observed expression changes of the indicated tRNA in vesicle fractions after benzonase treatment was not changed except tRNA^Val AAC^ (Fig. 2e) although the pattern of tRNA^Val AAC^ in quantification remains similar. Under the same benzonase treatment, plasmid DNA as control was completely degraded (Supplemental Fig. S2d). All together, these data suggest that select tRNAs, but not all, are loaded in extracellular vesicles for their release through urinary tract, and release of distinct extracellular tRNAs are modulated during kidney injury.

### Extracellular and intracellular distributions of vesicle-loaded tRNA^Glu^ under oxidative stress

**Figure 3:**
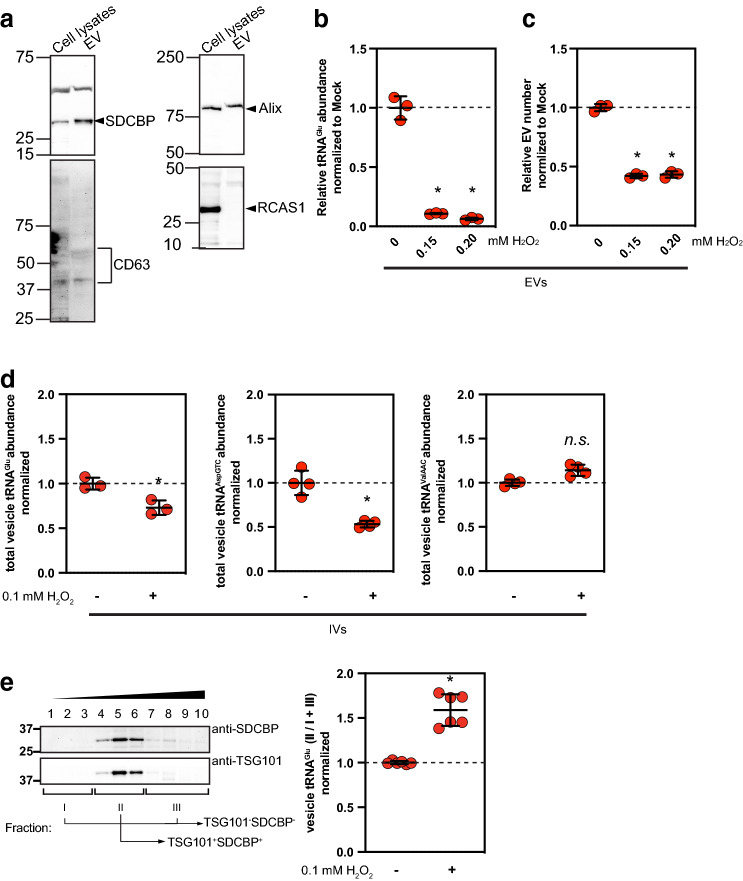
Extracellular and intracellular distributions of vesicle-loaded tRNA^Glu^ under oxidative stress. BUMPT cells were treated with the indicated concentrations of H_2_O_2_ for 1 h and thereafter conditioned for 23 h with serum-free, H_2_O_2_-free growth media. Note that cell viability was not significantly affected in this condition. (**a**) Immunoblotting analysis showing the indicated proteins in extracellular vesicle (EV) fraction, validating the extracellular vesicle isolation protocol used in this study. RCAS1 was used as negative control. (**b**) Relative tRNA^Glu^ abundance in extracellular vesicles from cells under oxidative stress. Benzonase was used to degrade RNAs associated nonspecifically with the surface of extracellular vesicles. *p < 0.05. (**c**) Total number of extracellular vesicles (EV) from cells under oxidative stress. Note that transient oxidative stress decreases release of extracellular vesicles. *p < 0.05. (**d**) Relative abundances of intracellular vesicle tRNA^Glu^, tRNA^AspGTC^, and tRNA^ValAAC^ under oxidative stress. Benzonase was used to degrade cytoplasmic RNAs associated nonspecifically with the surface of intracellular vesicles. Note that transient oxidative stress resulted in reduced loading of tRNA^Glu^ and tRNA^AspGTC^ in intracellular vesicles. *p < 0.05. (**e**) Transport of tRNA^Glu^ to SDCBP or TSG101-positive endosomes under oxidative stress. A 0.4–1.8 M continuous sucrose gradient was used to separate intracellular vesicles enriched with exosome cargo proteins, SDCBP and TSG 101. Post-nuclear supernatants were pelleted at 100 K × g, and the resuspended pellets were top-loaded on the gradient to separate intracellular vesicles, based on their size and density. Note that benzonase was used to degrade cytoplasmic RNAs. Total RNA measurement, RT-qPCR, and western analysis were performed as described in “Methods” section. *p < 0.05.

Ischemia–reperfusion (IR) is known to produce reactive oxygen species in kidney tissues which is a major injury response^29–32^. To model extracellular vesicle-based tRNA release in response to ischemic injury and delineate the underlying molecular mechanism, we challenged BUMPT cells, which are derived from mouse renal proximal tubules^33^, with oxidative stress (Fig. 3). BUMPT monolayers were treated with the indicated concentrations of H_2_O_2_ for 1 h, and H_2_O_2_-containing media were then replaced with fresh growth media to incubate for 23 h. After the incubation, the conditioned media were collected to isolate extracellular vesicles. Immunoblotting analysis showed that SDCBP, Alix and CD63, three extracellular vesicle markers were present in the extracellular vesicle fraction from the conditioned media (Fig. 3a). In contrast, marker analysis with RCAS1 showed that the extracellular vesicle fraction was not contaminated with Golgi compartments^34^. Compared with the control condition, this challenge decreased tRNA^Glu^ in extracellular vesicles (Fig. 3b). The observed tRNA release to extracellular milieu could be due to either regulated release of extracellular vesicles, number *(biogenesis)* or selective sorting of tRNAs to internal endosomes destined to extracellular vesicles *(loading)*, in addition to transcriptional regulation. Does a lower number of extracellular vesicles, including exosomes released in the biological fluids lead to a reduction of tRNAs in extracellular milieu? Or does decreased tRNAs that transit the endocytic pathway lead to a reduction of extracellular tRNAs? To distinguish these possible mechanisms, we measured total numbers of extracellular vesicles (EV) under the same condition used in Fig. 3a. Consistent with the observed levels of tRNAs in extracellular vesicles, the number of the vesicles from BUMPT monolayers treated with H_2_O_2_ was significantly decreased, compared with that from control (Fig. 3c). Thus, the expression pattern of tRNAs from cells treated with H_2_O_2_ for 1 h is accounted for by decreased release of extracellular vesicles from the cell surface. Next, in order to quantify loading of tRNAs to endosomes from the cytoplasm, BUMPT cell lysates were treated with benzonase to eliminate cytosolic nucleic acid, including tRNAs, spun to collect membrane fractions, and then fractionated with a sucrose density gradient. Quantification of nuclease-resistant, extracellular vesicle enriched tRNA^Glu^ and tRNA^AspGTC^ were significantly reduced under the oxidative stress while tRNA^ValAAC^ was not changed in abundance (Fig. 3d), indicating that oxidative stress perturbs loading of a specific tRNA set into membrane vesicles. Interestingly, among these tRNAs, the abundance of tRNA^Glu^ in membrane vesicles enriched with exosome markers, TSG101 and SDCBP (also called syntenin)^35^ in the density gradient, normalized to those not loaded with TSG101 and SDCBP was significantly increased after H_2_O_2_ treatment (Fig. 3e). It is highly likely that these membrane vesicles are endosomes, though we cannot completely rule out other vesicles packaging tRNAs with similar densities. Because TSG101 and SDCBP are well-studied endosome-located cargoes released through exosomes when they are released extracellularly^35, 36^, this result is likely indicative of loading of tRNAs to exosomes-destined endosomes. Taken together, these data suggest oxidative stress to BUMPT cells perturb vesicle trafficking of tRNAs, linked to release of extracellular vesicles, including exosomes.

### Maf1-mediated transcriptional repression in extracellular release of vesicle-loaded tRNA^Glu^ under oxidative stress

**Figure 4:**
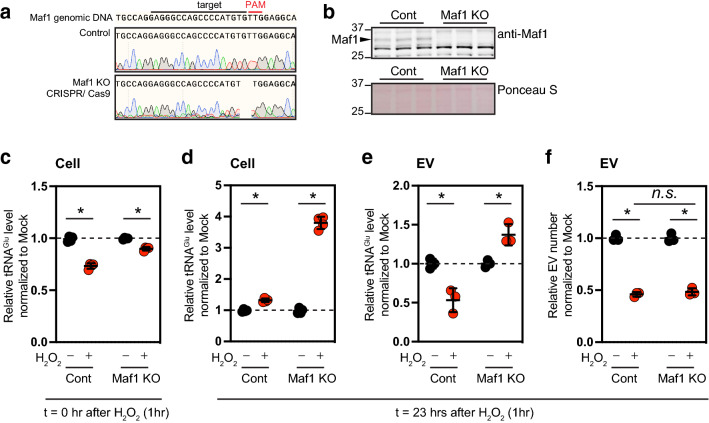
Maf1-mediated transcriptional repression in extracellular release of vesicle-loaded RNA^Glu^ under oxidative stress. (**a**) Sanger sequencing of the Cas9/CRISPR-targeted Maf1 locus. The sequencing chromatograms of sgRNA target region indicate homozygous status of the Maf1 locus in Maf1 knockout (KO) cells. (**b**) Immunoblotting analysis with anti-Maf1 confirms the loss of Maf1 expression in BUMPT cells. Ponceau S staining was used to determine equal amounts of proteins loaded per lane. (**c**–**e**) Changes in abundance of intracellular (cell) and extracellular vesicle (EV) tRNA^Glu^ in control and Maf1 knockout (KO) cells treated with 0.1 mM of H_2_O_2_ at the indicated timepoints. To collect conditioned media after oxidative stress, cells were treated with H_2_O_2_ for 1 h and thereafter exposed to serum-free, H_2_O_2_-free growth media for 23 h. Expression levels of tRNA^Glu^ were normalized to total RNA amount, and then relative expression values of tRNA^Glu^ of treated group normalized to control group were plotted. Because transient oxidative stress resulted in significant changes in total RNA levels and U6 snoRNA (not shown), relative expression of tRNA^Glu^ was normalized in this manner (cell number and conditioned media volume). For extracellular vesicle samples, benzonase was treated as described in “Methods” section. (**f**) Relative number of extracellular vesicles from control and Maf1 knockout (KO) cells under oxidative stress. Cells were treated as in (**c**–**e**). Note that transient oxidative stress decreases release of extracellular vesicles (*number*) regardless of the status of Maf1 expression.

Because Maf1 has been reported as a key repressor of tRNA transcription^37, 38^, we tested whether Maf1 regulates the abundance of intracellular tRNAs and consequently affects extracellular release of tRNAs under oxidative stress. For this experiment, we generated a Maf1 knockout (KO) cell line, using CRISPR/Cas9 technology, as performed in our previous works^39^. Sanger sequencing of genomic DNA (Fig. 4a) and immunoblotting analysis (Fig. 4b) confirmed inactivation of the target Maf1 gene and its expression by the NHEJ strategy. The indicated BUMPT monolayers were treated with H_2_O_2_, as described in Fig. 3 and then total RNAs in cell lysates and extracellular vesicles from the conditioned media were isolated at the indicated timepoints. Both the abundance of tRNA^Glu^ from control and Maf1 KO cells 23 h after H_2_O_2_ treatment were increased, and furthermore the abundance of tRNAGlu in Maf1 KO cells showed a remarkable increase, compared with that in control cells, in response to oxidative stress. (Fig. 4d). In contrast, the intracellular tRNA^Glu^ in the total cell lysates of control and Maf1 KO cells treated with H_2_O_2_ for 1 h were decreased (Fig. 4c). These data suggest that Maf1 exerts a strong transcriptional repression activity after the 1-h stress. Next, to understand how complete depletion of Maf1 affects extracellular vesicle-loaded tRNA^Glu^ under oxidative stress, we measured the abundance of tRNA^Glu^ as well as the number of extracellular vesicles in the conditioned media. In contrast to decreased abundance of extracellular vesicle tRNA^Glu^ from control cells, which is reproducibly shown in Fig. 3, tRNA^Glu^ abundance in extracellular vesicles was increased (Fig. 4e). Both Maf1 KO and control cells showed similar decreased release (number) of extracellular vesicles under oxidative stress, suggesting expression of Maf1 does not alter release rate of extracellular vesicles under the stress (Fig. 4f). Taken together, these findings indicate that Maf1 transcriptionally represses tRNA^Glu^ expression during recovery following oxidative stress, which results in decreased abundance of tRNA^Glu^ in extracellular vesicles along with decreased release of extracellular vesicles.

### m^1^A Methylation of intracellular and extracellular vesicle-loaded tRNAs under oxidative stress

**Figure 5:**
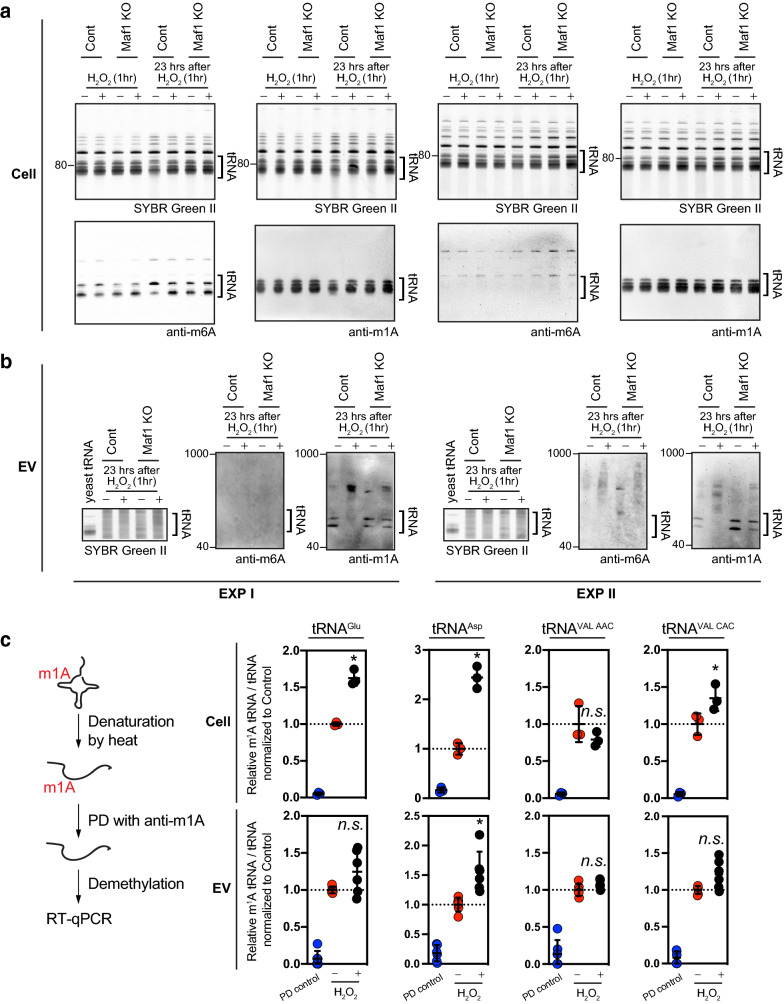
m^1^A methylation of intracellular and extracellular vesicle tRNAs under oxidative stress. Base methylation of intracellular (cell, **a**) and extracellular vesicle (EV, **b**) RNAs in BUMPT, control and Maf1 knockout cells under oxidative stress. Equal amounts of small RNAs (≤ 200 nts) for intracellular tRNAs or total RNAs of extracellular vesicle tRNAs from the indicated treatment were separated under denaturing conditions. For extracellular vesicle-loaded tRNAs, benzonase was treated as described in “Methods” section. The membranes were probed with the indicated antibodies to detect base modifications. The size of tRNA (]) was marked based on purified yeast tRNAs. Note that equal amounts of RNAs were loaded to detect the differences of degree in the indicated modifications and SYBR green II stain showed similar amounts of RNAs loaded per lane. The m1A MeRIP-RT-qPCR analysis (**c**) with intracellular and extracellular vesicle tRNAs at the indicated conditions. The relative ratios of m^1^A methylated to total (methylated and non-methylated) tRNAs as input calculated and normalized to the value from control (no H_2_O_2_) were plotted. *PD* Pulldown. *p < 0.05. *n.s.* non-significant.

tRNAs are heavily modified at their nitrogenous base, and the modification, including methylation, is known to play key roles in stabilizing their tertiary structure and fine-tuning in the codon-anticodon interaction^24, 40^. For example, N1-methyladenosine, m^1^A is required for functional tRNAs^41^ and used to detect conformational changes of tRNAs, which was shown to be much more rapidly represent cellular stress, compared with other apoptotic and DNA damage markers^42^. To test if oxidative stress alters base methylation, and altered methylation is associated with cargo selection for extracellular vesicles, we first determined base methylations of small RNAs in cells and extracellular vesicles. For cellular RNAs, small RNAs with shorter than 200 nucleotides were isolated, and the indicated base methylations were then analyzed by RNA blots using the antibodies recognizing N6-methyladenosine (m^6^A) and N1-methyladenosine (m^1^A). Equal amounts of RNA were separated on the blots, as shown by the SYBR Green II stain. While m^6^A modification of small RNAs spanning 40 bps and 200 bps was not changed at the indicated experimental conditions, m^1^A modification showed a slight fluctuation in the small RNAs from cell lysates, toward an increase under oxidative stress (Fig. 5a). Next, both m^1^A and m^6^A modification of RNAs present in extracellular vesicles were measured (Fig. 5b). For this measurement, total RNAs loaded in extracellular vesicles were used, due to a limited RNA amount at the same experimental scale. m^6^A methylation of small RNAs loaded in extracellular vesicles was not detectable although m^6^A methylation of cellular small RNAs in cells was recognized by the same antibody. Because this could be from low sensitivity of the antibody used in the study, we were unable to make a conclusion on m^6^A methylation of small RNAs loaded under oxidative stress. In contrast, m^1^A modification of small RNAs under the stress was clearly decreased in extracellular vesicles, regardless of Maf1 expression, indicating m^1^A hypo-methylated, small RNAs released via extracellular vesicles after oxidative stress.

Next, to test if the observed m^1^A methylation is directly associated with intracellular and extracellular vesicle tRNAs, we performed m^1^A MeRIP (m^1^A methylation RNA immunoprecipitation) and following RT-qPCR analysis. Total RNAs isolated from cell lysates and extracellular vesicles were heat-denatured to expose m^1^A and subjected to immunoprecipitation using anti-m^1^A. Both the input RNA for immunoprecipitation and the eluted m^1^A-containing RNAs from the pulldown were demethylated and then converted to cDNA to determine the abundance of the indicated tRNAs using qPCR (Fig. 5c). The m^1^A MeRIP-RT-qPCR analysis showed that oxidative stress increased the relative ratio of m^1^A methylated tRNAs: tRNA^Glu^, tRNA^Asp^, and tRNA^ValCAC^ to total intracellular tRNAs in each experiment which is normalized to the ratio measured in control condition. This is consistent with the slight increase of m^1^methylation of intracellular tRNAs observed in RNA blot. However, for tRNA^Glu^, tRNA^ValAAC^, and tRNA^ValCAC^ loaded in extracellular vesicles, oxidative stress did not change the ratio of m^1^A methylation while the oxidative stress increased the methylation of tRNA^Asp^. This data suggests altered methylation of tRNAs within cells is not associated with cargo selection for extracellular vesicles in response to oxidative stress. Probing m^1^A methylation of tRNAs released via extracellular vesicles might not be useful to monitor cellular stress.

## Discussion

Extracellular vesicles grab great attention from basic biology disciplines to translational and industrial application. The use of extracellular vesicles has been recently proposed as a way to noninvasively measure kidney injury^14, 43, 44^ and prevent worsening renal function presumably through intercellular communication^11^. Given these recent studies, we addressed if extracellular release of vesicle-enclosed tRNAs responds to kidney injury and how such tRNAs traffic intracellularly and are transported to the extracellular space under oxidative stress.

In this study, we found that renal ischemic injury resulted in a dramatic decrease of select tRNAs in urinary vesicles at the injury state and then gradual return to the pre-injury state during the recovery state. Theses observed changes of tRNA abundance in urinary vesicles are likely to show an initial response to the injury and thereafter adaptation following the injury. To validate the RNA-sequencing data, we examined U6 snoRNA as a single reference gene and found that the abundance of U6 snoRNA in the urinary vesicles from sham and injured animals at the 24-h timepoint after surgery was not significantly affected. Next, to establish a robust quantification assay, we compared several qPCR protocols by adopting the demethylation strategy combined with different priming methods. Using the optimized amplification and normalization condition, we validated the expression patterns of isoacceptor tRNAs: tRNA^Ala^, tRNA^Glu^, tRNA^Asp^, tRNA^Val^ in urinary vesicles from sham and injured animals. A recent publication by Tosar and Cayota et al*.* showed that extracellular vesicle-depleted fraction from cultured cell media is enriched in fragmented tRNAs, due to RNase activities in the extracellular space while extracellular vesicles contain non-fragmented tRNAs^45^. Consistent with this finding, our data confirms that non-fragmented tRNAs from urine are predominantly present in the extracellular vesicle fractions as our detection primer sets are designed to bind to the near ends of mature tRNAs and enable to amplify full-length sequences of tRNAs. Many RNAs in biological fluids bind or associate nonspecifically to the surface of extracellular vesicles and could be contributed to their presence in extracellular vesicle composition, which might be a threat to reproducibility in their quantitation. To make sure, the differentially released tRNAs are encapsulated in membrane vesicles, we further tested their susceptibility to a nuclease, Benzonase. Given that the enrichment in the membrane fraction and protection from the nuclease treatment, we concluded select full length tRNAs released to urine are predominantly loaded in extracellular vesicles.

Depending on the origins of extracellular vesicles, the observed tRNA release to extracellular milieu could be regulated at multiple steps: loading to intracellular vesicles; intracellular vesicle traffic; and release from the cell surface, including endosome fusion to the plasma membrane (exosomes) and shedding of the plasma membrane (ectosomes). Our data demonstrate that extracellular release of tRNAs is controlled at the multiple levels under oxidative stress. These include a limited vesicle loading of tRNAs, increased transit of vesicle-loaded tRNA to endosomes destined to extracellular vesicles, and blockade of release of extracellular vesicles from the cell surface. A number of studies have established that select tRNAs are transported to the nucleus from the cytoplasm in response to multiple stress challenges, including deprivation of amino acid and glucose^46^, heat^47^, and oxidative stress^23^. Stress-induced retrograde tRNA nuclear import is considered as a cellular defense mechanism that represses global protein translation. A crucial addition by our data is to stress-responsive tRNA traffic to extracellular space, a new trafficking route of tRNAs. Oxidative stress perturbed cytoplasmic tRNAs to endosomes and shifted vesicular tRNAs to late endosomes carrying SDCBP or TSG101, but these endosomes were unable to be released extracellularly. However, understanding how the new traffic contributes to cellular function of tRNAs would require further experimental validation at this moment.Given that tRNA retrograde nuclear transport limits cytoplasmic availability of tRNAs, it would be interesting to know if exosomal (extracellular vesicle) release of tRNAs under oxidative stress also serve as a defense mechanism that curtails harmful translation during oxidative stress as both nuclear and extracellular pathways can limit the availability of cytoplasmic tRNAs. Alternatively, it is also possible that this traffic route is used to compartmentalize or dump damaged or improperly modified tRNA to the extracellular space or save functional tRNAs within cells as a recovery step from oxidative stress.

Because tRNA availability as cargo can affect the abundance of tRNAs in extracellular vesicles, we completely inactivated maf1 expression, a master chronic transcriptional repressor of tRNAs and examined the effect of this regulator in extracellular release of tRNAs. As expected, complete depletion of Maf1 expression did not alter release of extracellular vesicles (*number*) from the cell surface. Maf1, however, exerts tRNA repression more prominently after oxidative stress, rather than during the stress because release of extracellular vesicle tRNAs was increased only at the recovery period following the oxidative stress when maf1 is completely inactivated. This data indicates that maf1-mediated transcriptional repression also contributes to the observed decrease of tRNAs in extracellular vesicles and as a result, cells tend to keep tRNAs intracellularly in response to oxidative stress.

Despite the presence of full length tRNAs in extracellular vesicles under oxidative stress, it is possible that release of these tRNAs via extracellular vesicles could be improperly modified. Considering the key structural role of m^1^A methylation in tRNA^41, 42^, we thus tested if immature (and hence not functional) tRNAs are released after oxidative stress.

Our m^1^A MeRIP-RT-qPCR analysis suggests that m^1^A methylation is unlikely to serve as a targeting signal of these membrane-enclosed tRNAs for their extracellular release under oxidative stress. However, there is a slight reduction in the relative ratio of m1A-methylated tRNAs loaded in extracellular vesicles to those in intracellular vesicles, indicating a weak propensity to keep these mature tRNAs within cells. Further investigation will be required to test this possibility more definitely.

In summary, our work shows that renal IR injury and oxidative stress decrease extracellular release of tRNAs. Oxidative stress mobilizes select, non-fragmented tRNAs to endosomes destined to extracellular vesicles and suppress biogenesis of extracellular vesicles, including exosomes. During the recovery following oxidative stress, Maf1 represses cellular tRNA expression. As an output, oxidative stress decreases extracellular release of vesicle-loaded tRNAs which are non-fragmented. Combined with m1A methylation status of isoacceptor tRNA abundance, this result not only adds a new layer of regulation to tRNA stress response but also demonstrates dynamic changes of tRNA abundance in urine over the progression of AKI that could be a valuable source for AKI diagnosis and prognosis.

## Methods

### Cell culture

BUMPT (Boston University Mouse Proximal Tubule clone 306), a mouse proximal tubule cell line^33, 48^ was maintained in DMEM with L-glutamine (Thermo) supplemented with 5% fetal bovine serum (Corning) and 100 U/ml of penicillin/streptomycin (Thermo). BUMPT cells were passaged in humidified 5% CO_2_ at 37 °C, at sub-confluence. Mycoplasma contamination of parental BUMPT cells used in this study was tested with LookOut mycoplasma PCR detection kit (Sigma). To collect the conditioned media, BUMPT cells were cultured for the indicated times, using serum-free medium, supplemented with L-glutamine and 100 U/ml of penicillin/streptomycin. For cell culture experiments, equal numbers of viable cells were plated after cell numbers were counted twice or three times, using Countess 3 FL Automated Cell Counter (Thermo) with Invitrogen viability test kit (Thermo).

### Animals and bilateral renal ischemia/reperfusion injury

All animal experiments were conducted in accordance with the National Institutes of Health *Guide for the Care and Use of Laboratory Animals* and the ARRIVE (Animal Research: Reporting of In Vivo Experiments) and were approved and monitored by the Augusta University Institutional Animal Care and Use Committee. Male rats (Sprague-Dawley, SD, 10 wks) were used from in-house colonies. Renal ischemia reperfusion surgery was performed as previously described^28^. Briefly, bilateral renal pedicles were exposed for clamping to induce 25 min of ischemia, and the clamps were then released to reperfuse with blood for 23–24 h. The sham operation was performed in the same way without clamping of the renal pedicles. Urine samples were collected and immediately stored at − 80 °C until used.

### Analysis of small RNA sequencing

The RNA sequencing data used for this study is available at the Gene Expression Omnibus database (GEO), GPL18694. The data was aligned to the reference genome mm10, and indexed using rsubread package^49^. Raw read counts of isoacceptor tRNAs were summed and data was median normalized using DEseq2 package^50^. The divergence of expression of tRNAs between sham and injured group for each time point was measured using Jensen-Shannon divergence.

### CRISPR/Cas9-mediated knockout of Maf1

Lentiviruses harboring the expression cassettes of a *Cas9-NLS* and a sgRNA targeting GAGGGCCAGCCCCATGTGT of *Maf1* genomic DNA sequence along with *pac*, a puromycin N-acetyltransferase as a drug selection marker were generated from Millipore Sigma. 18 h after plating, BUMPT cells were infected with the viral supernatant in 10 multiplicity of infection (MOI). 16 h after viral infection, cells were then cultured back in fresh normal growth medium for 24 h and thereafter selected with 1.5 μg/ml puromycin (Thermo) for 1 week. Each puromycin-resistant colony was cloned out to test complete inactivation of endogenous Maf 1 expression by indel mutations. Genomic DNAs from 25 clones were extracted using TRIzol (Thermo), and a 272-bp region flanking the *Maf1* double strand break site was PCR-amplified using Q5 High-Fidelity DNA polymerase (NEB). The following primers were used to amplify the 272-bp genomic DNA region: GAGACTGGAGATGCCCATATT (forward) and GTAGTCTCCACCTCAGCTTAC (reverse). Automated Sanger DNA sequencing verified three clones carrying the desired mutations for *Maf1* inactivation.

### RNA extraction from extracellular vesicles

For total RNA isolation from urinary extracellular vesicles, collected urine was immediately stored at − 80 °C until used. 1.5 ml of frozen samples were thawed at room temperature and centrifuged sequentially at 400 × g for 10 min at 25 °C, 4000 × g for 15 min at 25 °C, and 16,000 × g for 20 min at 25 °C. Next, the post-microvesicle supernatants were centrifuged at 200,000 × g for 75 min at 25 °C. The resulting pellets were resuspended with RNA lysis buffer (NEB or Zymo Research), and total RNAs were isolated using Monarch total RNA miniprep kit (NEB) or Quick-RNA Microprep kit (Zymo Research). To de-acylate tRNAs, Tris–HCl, (pH 9.0) buffer was added to isolated RNAs at the final concentration of 20 mM. The mixtures were incubated at 37 °C for 40 min. After the incubation, the pH of the mixtures was adjusted to 7.5. Similarly, for total RNA isolation from extracellular vesicles of the conditioned media from BUMPT cells, collected media were sequentially spun at 400 × g for 10 min at 4 °C, 4000 × g for 15 min at 4 °C, and 16,000 × g for 20 min at 4 °C. The supernatants were filtered through 0.2 μm PES syringe filter (Fisher Scientific) and then pelleted at 200,000 × g for 75 min at 4 °C. The resulting pellets were processed as described above.

To test benzonase susceptibility, pellets from sequential centrifugations described above except the final spin of 100,000 × g were carefully resuspended with 5 mM HEPES (pH 7.4) supplemented with 3 mM MgCl_2_ in order to avoid potential damages in extracellular vesicles and incubated with 100 unit/ ml Benzonase for 3 min at room temperature. The digestion reaction was terminated by adding the final concentration of 20 mM EDTA. As positive control, 500 ng of plasmids (10 kb) were digested with 50 unit/ml Benzonase for 3 min at room temperature.

### Sucrose gradient centrifugation

Cells were resuspended in a hypotonic buffer, 10 mM HEPES (pH 7.4)**/**5 mM EDTA**/**protease inhibitor cocktail (Thermo) and ruptured with a Dounce homogenizer. For benzonase treatment experiments, cells resuspended in 10 mM HEPES (pH 7.4)**/**1 mM MgCl_2_**/**protease inhibitor cocktail (Thermo)**/**25 U/ml of Benzonase (Millipore Sigma), were homogenized and then EDTA was added. The post-nuclear supernatants spun at 4000 × g for 15 min were then centrifuged at 200,000 × g for 75 min at 4 °C to pellet membrane fractions. The resulting pellets re-suspended with the hypotonic buffer were top-loaded on a 0.4–1.8 M gradient and subsequently centrifuged at 200,000 × g for 21 h at 4 °C. Ten fractions were collected from the top of the gradients. The perturbation of collected fractions were monitored using fluorescent density microspheres (Amersham). One-tenth of each fraction was subjected to total RNA isolation for RNA quantitation or protein preparation for SDS-PAGE and immunoblotting. Immunoblotting and RT-qPCR were performed as described below. Total RNAs were measured with Synergy LX Multi-Mode reader equipped withTake3 micro-volume plate (BioTek).

### Immunoblotting analysis

Proteins in fractions from sucrose gradient centrifugation were denatured by adding 4 × LDS sample buffer (Thermo) supplemented with DTT, were separated by SDS-PAGE, and subsequently transferred electrophoretically to nitrocellulose membrane. The blots were probed with the indicated primary antibodies followed by HRP-conjugated secondary antibodies for chemiluminescence detection. Unsaturated signals on the blot were capture with iBright FL1000 (Thermo). The primary antibodies used in this study are: anti-SDCBP (1:1000, Bio-Rad), anti-TSG101 (1:500, Santa Cruz Biotechnology), anti-Alix (1:1000, Cell Signaling Technology), anti-aquaporin1 (AQP1, 1:000, Santa Cruz Biotechnology), anti-RCAS1 (1:1000, Cell Signaling Technology), anti-CD63 (1:250, DSHB) and anti-Maf1 (1:250, GeneTex). Full-length blots are included in Supplementary Fig. S3.

### qPCR-based tRNA quantification

Extracted RNAs were treated with demethylase cocktails (Arraystar), according to the manufacturer’s instruction and were then cleaned up with Quick RNA microprep kit (Zymo Research). Next, poly (A) tailing of demethylated RNAs was performed with E. coli poly (A) polymerase (NEB) at 37 °C for 30 min. Demethylated, poly (A)-tailed RNAs were reverse transcribed with anchored oligo d(T) primers, d(T)_23_VN (IDT), using ProtoScript II reverse transcriptase (NEB). SYBR Green-based, real-time quantitative PCR (qPCR) detection (NEB) was performed using CFX96 touch PCR machine (Bio-Rad) or QuantStudio™ 6 Pro (Thermo). The primers used in this study were listed in Supplementary Fig. S2.

### RNA blot analysis

RNAs smaller than 200 nts were isolated using spin-column technology (Quick-RNA Microprep kit, Zymo Research). Equal amounts of isolated small RNAs or total RNAs per lane were run on TBE-Urea gel after heat denaturation, electrophoretically transferred to positively charged nylon membrane Hybond-N + (Amersham), and then cross-linked using UV irradiation (UV crosslinker CL-1000, UVP). After blocking with bovine serum albumin, the blots were probed with the indicated primary antibodies followed by HRP-conjugated secondary antibodies for chemiluminescence detection. To detect RNAs, SYBR Green II staining was performed according to the manufacturers’ instruction (Thermo). Unsaturated signals from chemiluminescence or fluorescence on the blots were captured with iBright FL1000 (Thermo). The primary antibodies used in this experiment are anti-m^6^A (1:5000, Thermo) and anti-m^1^A (1:5000, Abcam). Full-length gels and blots are included in Supplementary Fig. S3.

### m^1^A MeRIP (methylated RNA immunoprecipitation)

Dynabeads protein A magnetic beads (Thermo) pre-equilibrated with RIP buffer (10 mM Tris–HCl pH 7.5, 150 mM NaCl, 0.1% NP-40) were blocked with 0.2% (w/v) RNase-free ultrapure bovine serum albumin (Thermo) in RIP buffer, and then tumbled with anti-m^1^A (abcam) at 4 °C overnight. Unbound antibodies were removed by extensive washing with RIP buffer. Sample RNAs were denatured at 94 °C for 5 min and immediately cooled on ice. Denature RNA and RiboLock RNase inhibitor (Thermo) were added to the antibody-magnetic bead mixtures, and the reaction mixtures were subsequently incubated on the rotor for 2 h at 4 °C. After incubation, the bound RNAs were extensively washed with RIP buffer and eluted with RNA lysis buffer supplied in Quick-RNA Microprep kit (Zymo Research). RNAs from the immunoprecipitants were isolated using Quick-RNA Microprep kit according to the manufacturer’s instruction.

### Nanoparticle tracking analysis

To measure the numbers of extracellular vesicles isolated from urine or conditioned media, nanoparticle tracking analysis was performed using Zetaview (Particle Matrix). Isolated extracellular vesicles were filtered through 0.2 μm PES syringe (Fisher Scientific) and then immediately injected to the Zetaview to avoid unwanted aggregation, and all procedures for size measurement were performed according to the manufacturer’s instruction.

### Statistical analysis

Statistical significance was assessed using unpaired Student’s t-test unless stated otherwise. *P* values < 0.05 were considered statistically significant. No sample size was calculated. No data were excluded unless experimental controls indicate failure in sample processing or experimental steps. Multiple biological replicates for each group from independent experiments were used. Technical replicates were not considered as independent samples except Supplementary Fig. S2.

## Supplementary Information

Supplementary Figure S1.

Supplementary Figure S2.

Supplementary Figure S3.

## Data Availability

The RNA-sequencing data (Accession number GPL18694) used for this study is from the Gene Expression Omnibus database (GEO, NIH). All other data are contained within the article.
