# Supplementary material for: Differential release of extracellular vesicle tRNA from oxidative stressed renal cells and ischemic kidneys
Source: Sci Rep. 2022 Jan 31;12:1646. doi: 10.1038/s41598-022-05648-3 (PMC8803936; doi:10.1038/s41598-022-05648-3)
Supplement: Supplementary file 1 — Supplementary Figure S1. [file 41598_2022_5648_MOESM1_ESM.pdf]

Supplementary Figure S1

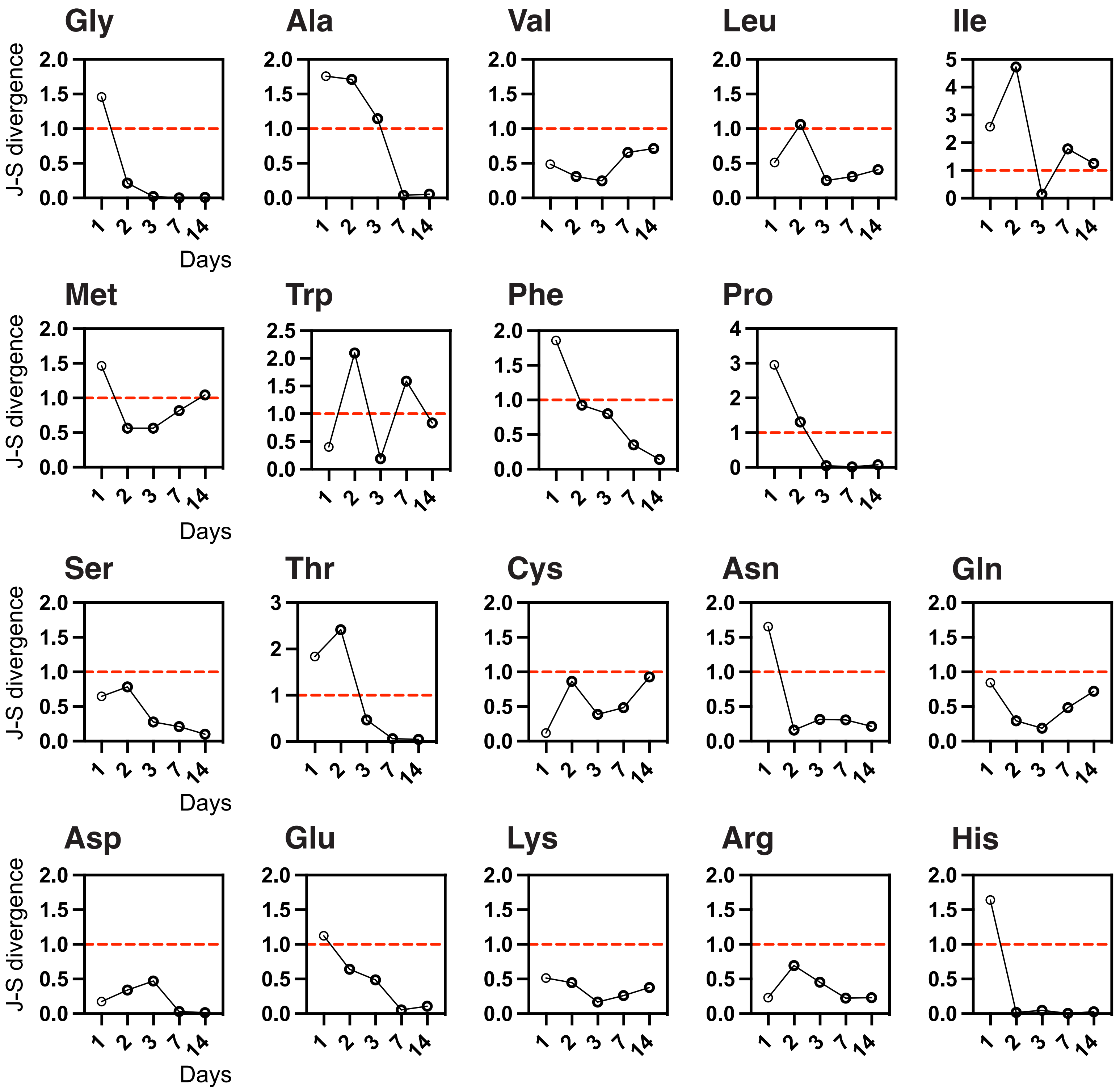

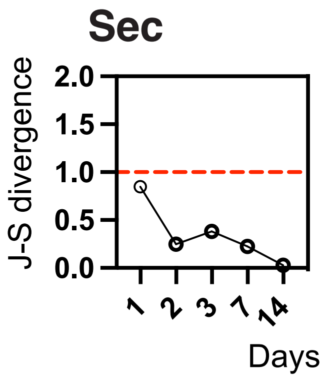

Supplementary Figure S1.
The association between recovery status and expression of tRNAs were shown through Jensen-Shannon divergence measures over time. Note that J-S divergence, a non-negative measure, converge to zero as expression level of tRNAs of injured group merge to that of control group. Most of the tRNAs showed overall negative trends suggesting recovery of tRNA profile over 14 days.
