# Supplementary material for: Differential release of extracellular vesicle tRNA from oxidative stressed renal cells and ischemic kidneys
Source: Sci Rep. 2022 Jan 31;12:1646. doi: 10.1038/s41598-022-05648-3 (PMC8803936; doi:10.1038/s41598-022-05648-3)
Supplement: Supplementary file 2 — Supplementary Figure S2. [file 41598_2022_5648_MOESM2_ESM.pdf]

Supplementary Figure S2

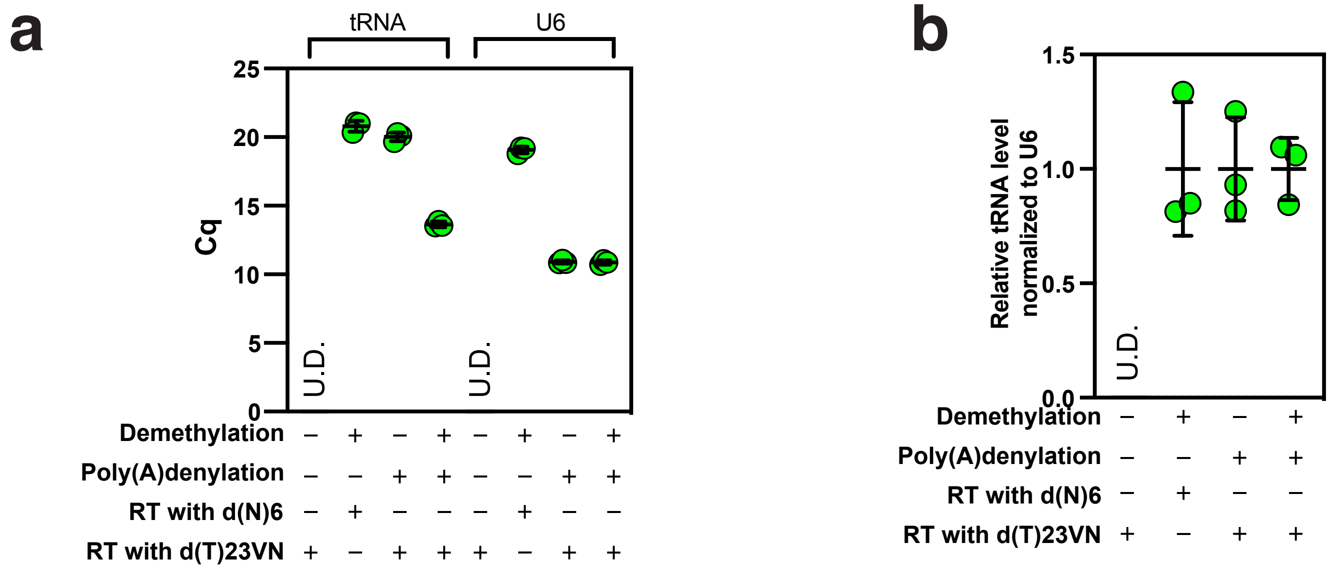

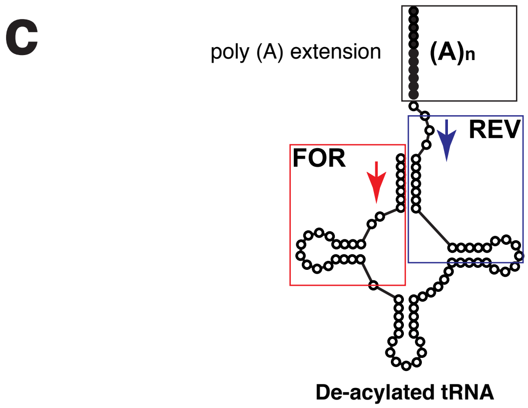

tRNA	Location	Sequence
ValAAC FOR<br>ValAAC REV	Ch11	TCCGTAGTGTAGTGGTTATCACG<br>GTTTCGAACCGGGGACCT
AlaCGC FOR<br>AlaCGC REV	Ch13	GGGGATGTAGCTCAGTGGTAG<br>ACCCGGGGCCTCATACAT
ValCAC FOR<br>ValCAC REV	Ch13	TGGTTATCACGTTCGCCTCA<br>GTTTCGAACCGGGGACCT
AlaCGC FOR<br>AlaCGC REV	Ch4	CCTGGTAGTCTAGTGGTTAGG<br>TTCCCTGACTAGGATTCAAAC
GluCTC FOR<br>GluCTC REV	Ch3	TGGTGGTCTAGTGGTTAGGA<br>CCTGACCGGGAATCGAAC
AspGTC FOR<br>AspGTC REV	Ch11	CCTCGTTAGTATAGTGGTG<br>CGGGGAATCGAACCC
GluCTC FOR<br>GluCTC REV	Ch10	TGGTGGTCTAGTGGTTAGGA<br>CCTGACCGGGAATCGAAC

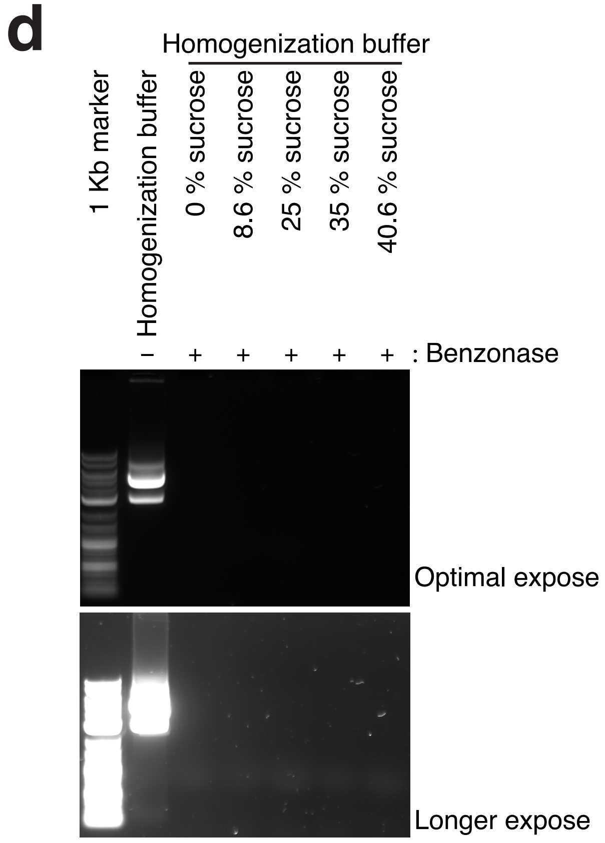

Supplementary Figure S2.

a) Comparison of qPCR-based tRNA amplification with demethylation and poly adenylation. The same amount of RNA isolated from extracellular vesicles of rat urine was tested with the combinations of demethylation, polyadenylation, and the indicated oligomer for reverse transcription. Data are from three technical replicates. Cq, quantification cycle. RT, reverse transcription. d(N)6, a random hexamer. D(T)23VN, an anchored oligo d(T). U.D., undetermined due to no amplification call. Note that deacylated tRNAs were used for all four conditions to compare tRNA quantification.

b) Comparison of tRNA quantifications of extracellular vesicles from urine samples of sham and 24 hours IRI with the indicated conditions. Data were normalized to U6 expression and are from three biological replicates. U.D., undetermined due to no amplification call. Note that deacylated tRNAs were used for all four conditions to compare tRNA quantification.

c) Primers used for RT-qPCR and their target locations.

d) Benzonase activities in different fractions from sucrose gradient centrifugation. Plasmid DNAs were added to the indicated sucrose/imidazole buffers and then treated with benzonase. One fifth of sample volumes was run on 1% agarose gel electrophoresis and stained with ethidium bromide to monitor degradation of plasmid DNAs
