# Supplementary material for: Differential release of extracellular vesicle tRNA from oxidative stressed renal cells and ischemic kidneys
Source: Sci Rep. 2022 Jan 31;12:1646. doi: 10.1038/s41598-022-05648-3 (PMC8803936; doi:10.1038/s41598-022-05648-3)
Supplement: Supplementary file 3 — Supplementary Figure S3. [file 41598_2022_5648_MOESM3_ESM.pdf]

Supplementary Figure S3

Figure 2a

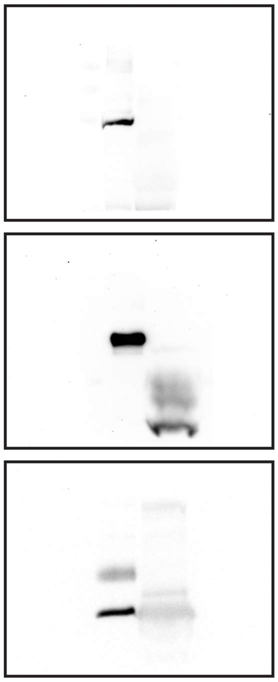

Figure 3e

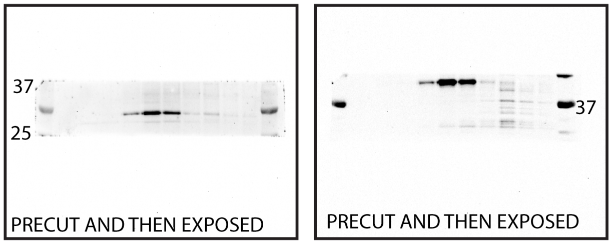

Figure 4b

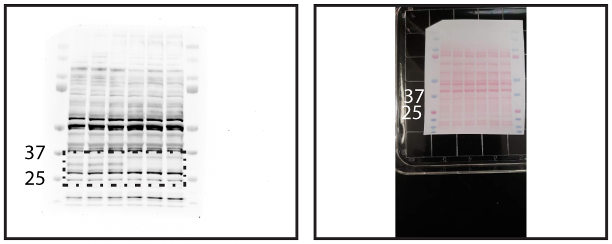

Figure 5

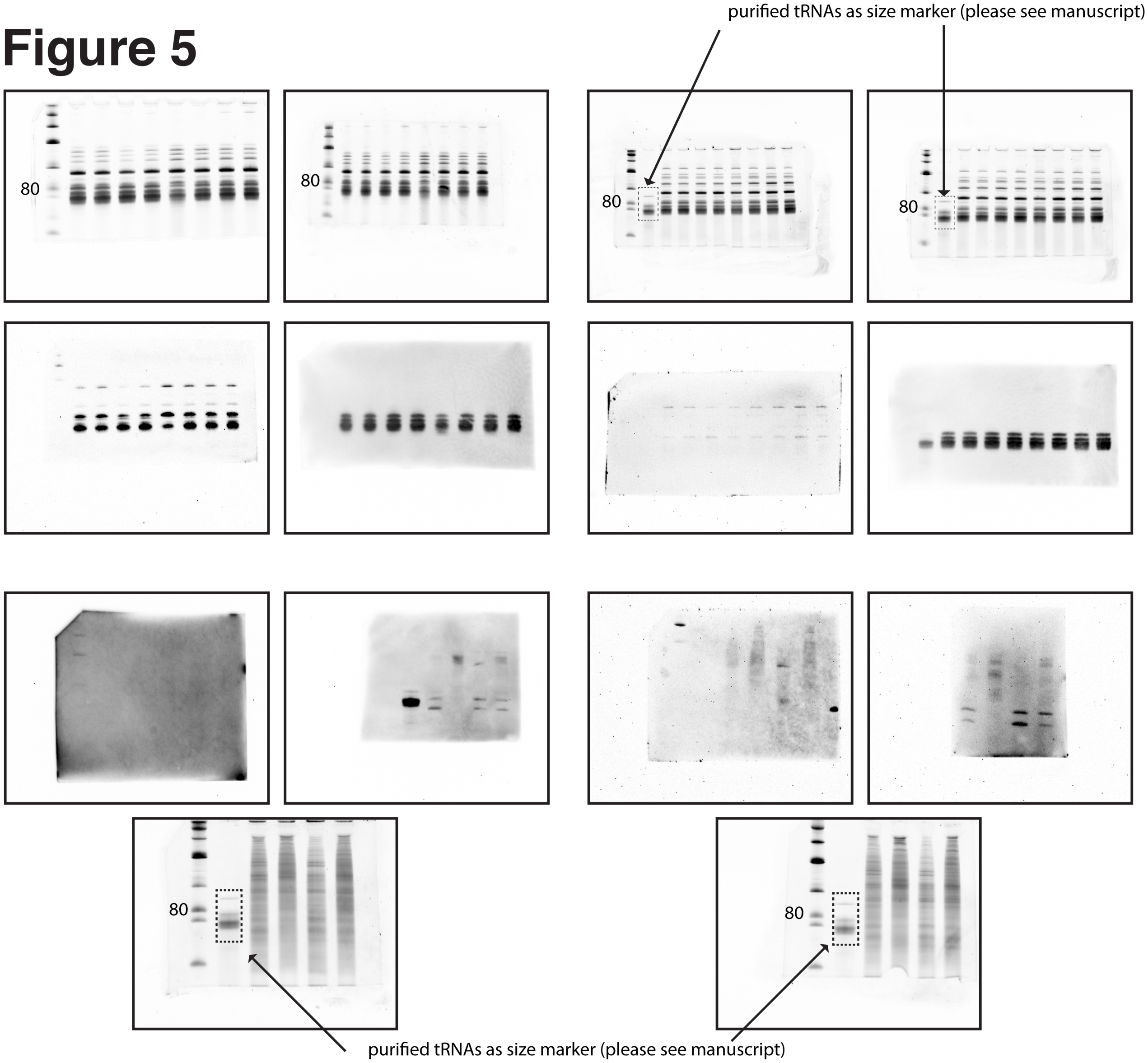

Figure 3a

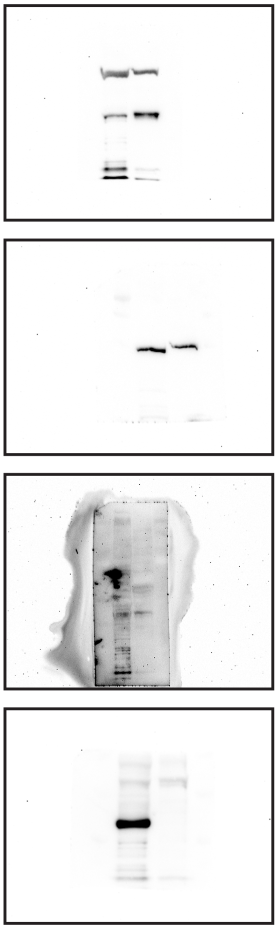

Figure S2d

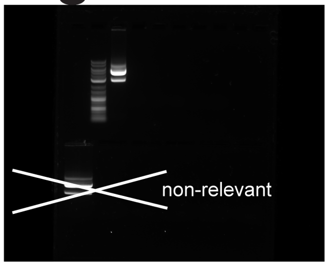

Supplementary Figure S3.
Full scan of cropped immunoblots. Uncropped scans of immunoblots of the indicated figures are shown.
